# Bioelectricity in dental medicine: a narrative review

**DOI:** 10.1186/s12938-023-01189-6

**Published:** 2024-01-03

**Authors:** Qingqing Min, Yajun Gao, Yao Wang

**Affiliations:** 1 Department of Endodontics, Wuxi Stomatology Hospital, Wuxi, 214000 China; 2 Department of Implantology, Wuxi Stomatology Hospital, Wuxi, 214000 China

**Keywords:** Bioelectricity, Electrophysiological, Dentistry

## Abstract

**Background:**

Bioelectric signals, whether exogenous or endogenous, play crucial roles in the life processes of organisms. Recently, the significance of bioelectricity in the field of dentistry is steadily gaining greater attention.

**Objective:**

This narrative review aims to comprehensively outline the theory, physiological effects, and practical applications of bioelectricity in dental medicine and to offer insights into its potential future direction. It attempts to provide dental clinicians and researchers with an electrophysiological perspective to enhance their clinical practice or fundamental research endeavors.

**Methods:**

An online computer search for relevant literature was performed in PubMed, Web of Science and Cochrane Library, with the keywords “bioelectricity, endogenous electric signal, electric stimulation, dental medicine.”

**Results:**

Eventually, 288 documents were included for review. The variance in ion concentration between the interior and exterior of the cell membrane, referred to as transmembrane potential, forms the fundamental basis of bioelectricity. Transmembrane potential has been established as an essential regulator of intercellular communication, mechanotransduction, migration, proliferation, and immune responses. Thus, exogenous electric stimulation can significantly alter cellular action by affecting transmembrane potential. In the field of dental medicine, electric stimulation has proven useful for assessing pulp condition, locating root apices, improving the properties of dental biomaterials, expediting orthodontic tooth movement, facilitating implant osteointegration, addressing maxillofacial malignancies, and managing neuromuscular dysfunction. Furthermore, the reprogramming of bioelectric signals holds promise as a means to guide organism development and intervene in disease processes. Besides, the development of high-throughput electrophysiological tools will be imperative for identifying ion channel targets and precisely modulating bioelectricity in the future.

**Conclusions:**

Bioelectricity has found application in various concepts of dental medicine but large-scale, standardized, randomized controlled clinical trials are still necessary in the future. In addition, the precise, repeatable and predictable measurement and modulation methods of bioelectric signal patterns are essential research direction.

**Graphical abstract:**

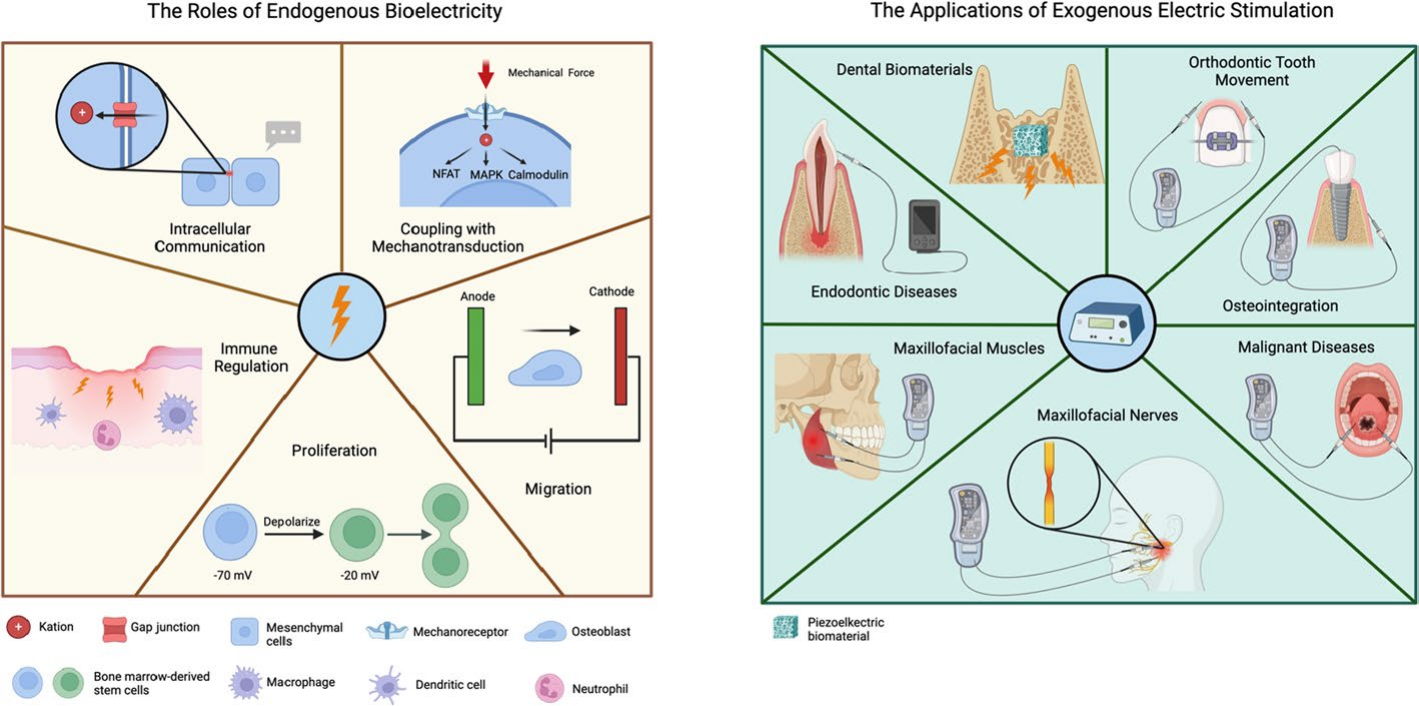

## Introduction

Bioelectricity is termed as any electrical phenomenon that is either actively generated by cells or applied to cells to influence their phenotype [1]. In 1792, Luigi Galvani was the first to record that an unintended spark discharge caused the contraction of frog muscle fibers. He asserted that the animal neuromuscular system operates as an electrodynamic system with the capacity to generate electricity. [2, 3]. The experiments on electric eel by Alexander von Humboldt in 1800s further refined Galvini’s theory [4]. In 1840s, du Bois-Reymond verified and developed techniques for recording the tiny electric currents generated during nerve conduction, involving the insertion of electrodes into animal tissues, which laid down the foundation of modern electrophysiology [5]. In the 1940s, electrical stimulation therapy was tried to be introduced in some clinical practices such as treatment of arrhythmia [6] and epilepsy [7]. But almost all these recordings are case reports, and massive severe complications and failure were reported due to limited electrophysiological knowledges [8–10]. After 1950s, commercialized electric defibrillator was invented [11] and electrical stimulation was confirmed to be useful for cardiac pacing [12]. Up to now, electrical therapies has been verified to be beneficial to neuromuscular pain [13, 14], neuromuscular recovery [15, 16], wound healing [17], and bone fracture healing [18].

**Fig. 1:**
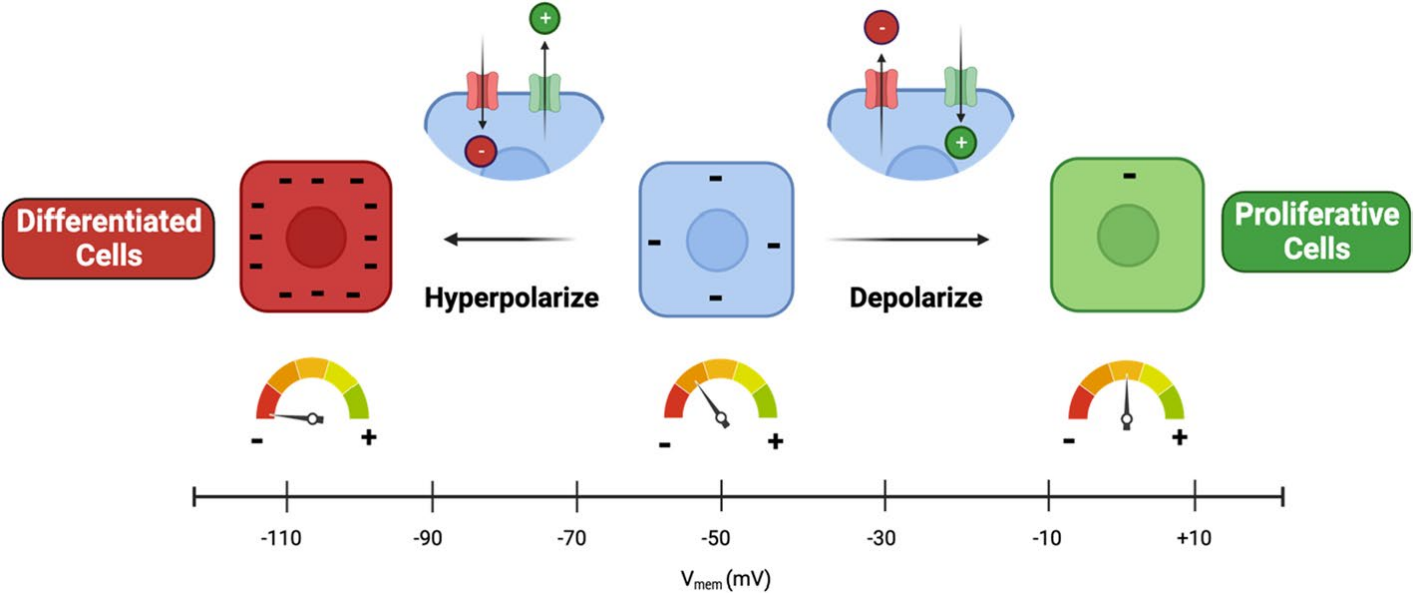
The schematic graphic displays how cells regulate transmembrane potential. Most cells maintain a transmembrane potential with the interior negatively charged relative to the exterior. When cell membrane receptors facilitate the influx of anions or efflux of cations, intracellular charge becomes more negative, a phenomenon referred to as hyperpolarization. Conversely, when anions efflux or cations influx, intracellular charge shifts towards less negative or even positive, termed depolarization

At present, bioelectrical studies encompass the measurement of voltage fluctuations and electric currents, as well as electrical interventions across a broad spectrum of scales, ranging from individual ion channel proteins to entire organs such as the heart. The transmembrane potential (V_mem_) is regarded as a fundamental aspect of endogenous bioelectricity. This phenomenon arises from the selective permeability of the cell membrane and the active transport facilitated by ion pumps, leading to distinct distributions of charged ions (e.g., Na^+^, K^+^, Ca^2+^, and Cl^−^) between intra- and extracellular compartments [19]. This constant imbalance of electric charge generates voltage differences between two sides of cytomembranes, termed transmembrane potential or V_mem_ [20]. In most cells, the resting transmembrane potential is negative on the inside relative to the outside; for instance, in neurons, the resting transmembrane potential is approximately − 70 millivolts (mV) [21]. However, this value is not static. When there is a rapid influx of positively charged ions (e.g., Na^+^) into the cell or the efflux of negatively charged ions (e.g., Cl^−^) out of the cell, the membrane becomes less negative or even positive, a phenomenon referred to as ‘depolarization.’ Conversely, when the transmembrane potential becomes more negative, it is termed ‘hyperpolarization’ (Fig. 1). These alterations in transmembrane potential play a crucial role in regulating electrical activities in excitable cells, such as neurons and muscle cells. Moreover, accumulated data have revealed the significant role of transmembrane potential in non-excitable cells [22]. Transmembrane potential is closely linked to the proliferation capacity of cells. In particular, rapidly proliferating embryonic and tumor cells tend to exhibit a reduced transmembrane potential difference, characterized by depolarization. Conversely, differentiated somatic cells, such as skeletal muscle cells, neurons, and fibroblasts, typically maintain a higher level of hyperpolarization [19]. The resting transmembrane potential of normal human breast epithelial cell is near − 60 mV but infiltrating ductal carcinoma tissue was found to be − 13 mV [23]. This probably due to aberrant expression of ion channels and transporters [24]. Behavsar et al. successfully manipulated the V_mem_ by blocking and unblocking charged ion transporting channels to affect cellular proliferation [19], which further verified the roles of transmembrane potential in cellular phenotype.

Moreover, numerous studies have demonstrated that external electrical stimulation (EStim) influences cellular behavior, including transmembrane potential shifts [25], differentiation of stem cell [26], cell proliferation [27], cell migration [28], inflammatory cytokines secretion [29] and collagen production [30]. However, the main limitation of current studies is the lack of consistency in the EStim parameters (type, duration, current, voltage, direction, etc.) they employed. It has been confirmed that EStim of different intensities probably lead to diverse, sometimes contrasting outcomes [25]. Thus, to date, there is no consensus regarding the optimal parameters for clinical EStim therapy. Our review provides an overview of the effects of endogenous bioelectricity and compiles a decade’s worth of research on external electric stimulation, including detailed parameters. Furthermore, we reviewed the applications of bioelectricity in the field of dental medicine and engage in a discussion regarding its prospects.

## Methods

The literature was searched using PubMed, Web of Science and Cochrane Library using the key words *bioelectricity*, *endogenous electric signal*, *electric stimulation* and *dental medicine.* Besides, the associated MeSH terms *dental medicine* was broadened to include *periodontology*, *orthodontics*, *implantology*, *endodontics*, *pediatric dentistry* and *maxillofacial surgery*. The BOOLEAN operators ‘AND’ and ‘OR’ were used to ensure maximum inclusion. Using PubMed database literature search strategies as an example, refer to Table 1.

**Table 1:** Search strategy used for PubMed database with MeSH subheadings

1	Bioelectricity
2	Endogenous electric signal
3	Electric stimulation
4	1 AND dental medicine
5	2 AND dental medicine
6	3 AND dental medicine

**Fig. 2:**
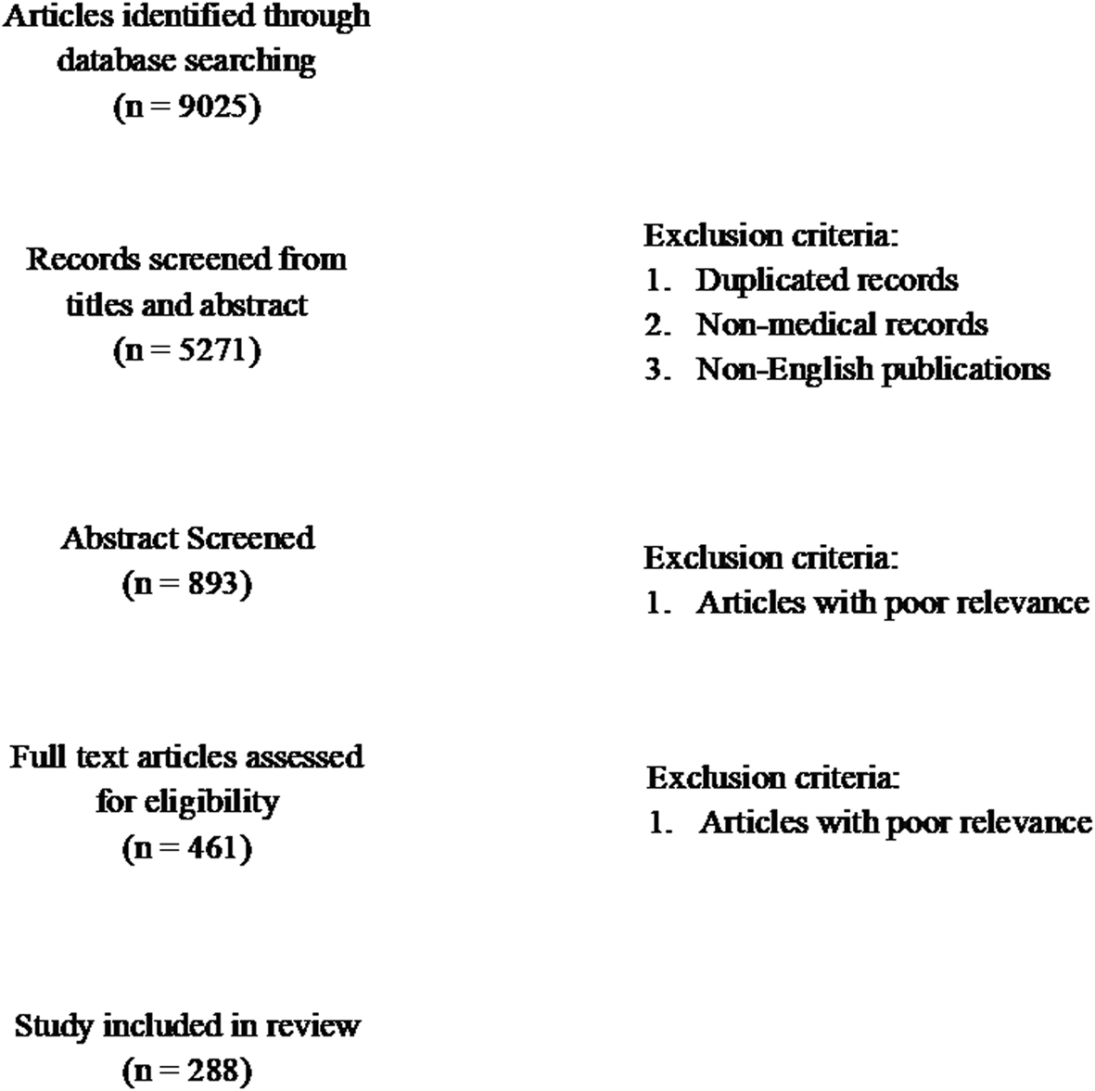
Flow diagram showing search process (following PRISMA guidelines)

A total of 9025 articles were retrieved. After removal of duplicated articles, non-medical papers, papers with poor relevance to the MeSH terms and non-English literature, 288 articles were scrutinized and discussed in this narrative review. Detailed search process and exclusion criteria can be found in Fig. 2. Each article was independently assessed by QM and one of the other authors, in cases where there was disagreement between researchers, consensus was reached by discussion between all the authors.

## Endogenous electrical signals

### The sources of bioelectricity: from organs to cells

The brain[31, 32], heart [33, 34], and bones [35] can generate endogenous bioelectricity in the human body. The brain is a remarkably electrically active organ that generates and conducts electrical signals through neurons. The excited state of a neuron responsible for signaling, known as the action potential (AP), is produced through the transmembrane transport of charged ions and transmitted as an electric signal along nerve axons [36]. It is noticeable that the nerve impulse has different action potential widths, frequencies and patterns which are essential to responses of downstream nerve cells or end-organs [37–39]. Besides, heart generates and propagates the electrical impulse to initiate and regulate its contraction and relaxation. The bioelectric impulse is generated from the sinoatrial node (SAN) and reaches the atrioventricular node (AVN), and activates ventricular myocardium through ventricular conduction system (VCS), this process has been completely reviewed by Weerd and Christoffels [40]. Interestingly, the bioelectric impulse also plays a role in the early formation of heart [41–43]. Wen et al. simulated the “funny” current, a current generated by the hyperpolarization-activated cyclic nucleotide-gated channel (HCN) family in the sinoatrial node (SAN) and crucially involved in the spontaneous diastolic depolarization of SAN cells, successfully promoting cardiogenesis in canine mesenchymal stem cells (cMSCs) [44, 45]. Bone and cartilage have piezoelectric property, for example, the human tibia generates a 300 μV piezoelectric potential from walking [46]. Bone surface compression induces a negative stress-generated potential (SGP), promoting tissue formation, while tensile forces generate a positive potential, leading to resorption [35]. In addition, when a fracture occurs, the fracture site turns more negatively charged. One plausible explanation for this phenomenon is that the negative potential may attract a greater number of cells to engage in the repair process [3, 35, 47]. The piezoelectric properties of bone tissue are not solely attributed to cellular activity but also stem from extracellular organic and inorganic compounds. For example, collagen exhibits piezoelectric effects especially when it is dry [35]. In the bone tissue, its water absorption is limited by another inorganic component, hydroxyapatite, known as another common piezoelectric material [48, 49]. Given that collagen has an abundance of electrons while hydroxyapatite has few, they are considered as the negative and positive electrodes in bone tissue, respectively [50].

From yeast to human stem cells, they all share a common ability to generate transmembrane potential by regulating specific ion channels, typically involving sodium, potassium, and calcium ions. This regulation leads to an imbalanced distribution of charged ions across the cell membrane. The action potential represents one of the earliest recognized processes associated with changes in transmembrane potential. In excitable cells, such as neurons and muscle fibers, external stimulation initially triggers the activation of voltage-gated sodium ion channels, resulting in the inward flow of sodium ions (Na^+^). This phenomenon induces cellular depolarization, causing a transient (< 1 ms) reversal of the transmembrane potential polarity [51]. Once the transmembrane potential reaches a critical level, known as the threshold potential, it triggers the subsequent initiation of an action potential through the activation of additional ion channels [52]. Within a brief duration of approximately 1 ms, the Na^+^ channel undergoes a conformational change, preventing further passage of Na^+^. Following this, K^+^ channels open, allowing K^+^ to flow out of the cell, leading to the restoration of the transmembrane potential towards negative values. This process is referred to as repolarization, marking the final phase of the action potential. In contrast, in non-excitable cells, changes in transmembrane potential also play a role in the activation of signaling pathways and the regulation of cellular metabolism, rather than being associated with nervous excitation or muscle contraction [53–55]. It is noteworthy that calcium and chloride channels play more essential roles in non-excitable cells than sodium or potassium [56, 57]. The following section outlines the activated mechanisms and effects of common ion channels:

#### The voltage-gated sodium channels (VGSCs)

The VGSC is a selective ion channel which generates rapid internal sodium ion influx that drive depolarization of cells [58] It was first described clearly as a fast-responding initiator of action potentials in neurons and other excitable cells by Hodgkin and Huxley in 1952 [59]. The sodium channel in eukaryotic organisms exhibits a high degree of conservation, composed of an α subunit with a noncovalently associated β1 subunit and a disulfide-linked β2 subunit [60, 61]. Sodium channel α subunits consist of approximately 2000 amino acid residues organized into four homologous domains, each containing six transmembrane segments. The β subunits, on the other hand, are composed of an N-terminal extracellular immunoglobulin-like fold, a single transmembrane segment, and a short intracellular segment. These β subunits play a crucial role in modulating the kinetics and voltage dependence of sodium channel activation and inactivation [62, 63]. The crystal structure of the active-state conformation of sodium channel has been revealed by Jian Payandeh et al. [64]. In addition, models of the resting-state conformation are also been obtained using all-atom molecular dynamics (MD) simulations [65]. These studies have demonstrated that the sliding helix mechanism, wherein the S4 segment maintains its helical conformation primarily as it moves along its long axis, is the most plausible model to explain the mechanism of voltage-dependent activation [66]. Interestingly, the short intracellular loop binding homologous domains III and IV of α subunit can fold into the intracellular mouth of the pore and blocking it to inactivate rapidly sodium channel, which is required for repetitive firing of action potentials in neural circuits [67]. The inactivation state of the sodium channel persists for a brief period to ensure the unidirectional propagation of the action potential. This temporary refractory period can also elevate the threshold for action potential initiation, resulting in reduced sensitivity to high-frequency stimulation [68]. However, a minor fraction of the total sodium current (approximately 1% to 2%) continues to flow even after cells return to their resting potential. This persistent sodium current is voltage-dependent and has been linked to atrial myopathy, although the precise mechanisms remain a subject of debate [69, 70]. Lastly, mutations of sodium channels cause numerous genetic diseases, including inherited forms of periodic paralysis, cardiac arrhythmia, epilepsy, and chronic pain [63, 71, 72]. Sodium channels blocker has also been used clinically as local anesthetics, antiarrhythmics, and antiepileptics [63].

#### The voltage-gated potassium channels (VGKCs)

Potassium channels are found in the cytomembrane of nearly all species, with a few exceptions among parasites, and VGKC is the most prevalent member within this family. VGKC is activated by a depolarized transmembrane potential and selectively induces K^+^ inward influx to promote repolarization [73]. Its fundamental structure consists of a tetramer, with each monomer containing one pore-forming domain. Collectively, these four pore-forming domains create a central pore through which ions are transported [74]. The activated and inactivated states of potassium channels are governed by two gates, each with distinct gating mechanisms: one located on the intracellular side and the other on the extracellular side. The coordinated operation of these two gates serves to establish a negative relationship, facilitating the transition of VGKC into the inactivated states [75]. Similar to sodium channels, VGKC also transitions into an inactivated state shortly after opening, a process linked to a conformational change in its ion selectivity filter (SF) [74]. Besides VGKC, which contributes to action potential generation and maintains synaptic transmission [76], ligand-gated (Kligand) channels are another crucial family member of potassium channels gated by chemical factors such as calcium ion concentration [76]. The existence of calcium-activated potassium supports that possible interaction between different ion channels. Anyway, in addition to regulate action potential, potassium channels were also confirmed to regulate tumor cell behavior, mitochondrial function and cell cycle [77–79].

#### The voltage-gated calcium channels (VGCCs)

The VGCC consists of α1, α2, β, γ, and δ subunits. It becomes active upon membrane depolarization and facilitates the influx of Ca^2+^ in response to both action potentials and subthreshold depolarizing signals [80]. The VGCC is mainly expressed on cardiac/smooth/skeletal muscular, endocrine, and immune cells [80–82]. Surprisingly, calcium current VGCC is detected in some primary tumor and breast cancer cell lines despite healthy human mammary epithelial cells do not express them [83, 84]. An explanation for this phenomenon is that the transient calcium influx through VGCC is essential for breast cancer cell growth, as the blockade of calcium channels significantly diminishes the proliferation of breast cancer cells [83]. Furthermore, based on the rate of activation, pharmacologic sensitivity, and also the voltage activation, VGCC can be categorized into six classes: T, L, N, P, Q and R types [85]. T and L-type are two of the most understood type of VGCC up to now [85]. T-type VGCC is characterized by lower levels of depolarization of activation, rapid inactivation and small single channel conductance. In contrast, L-type is only activated by a more positive transmembrane potential and has slower voltage-dependent inactivation [80, 85]. In addition to responding to action potentials and subthreshold depolarizing signals, calcium channels play a crucial role in coupling excitation and contraction in cardiac muscle. The influx of Ca^2+^ activates downstream ryanodine receptor 2 (RyR2), initiating release of Ca^2+^, which in turn activates actomyosin and leads to cardiac contraction [86]. Calcium ion also can regulate genes transcription and cellular secretion as a second messenger [87, 88].

#### The voltage-gated chloride channel (VGCLC)

Chloride channels (CLCNs) are extensively distributed in tissues and organs throughout the body, and they are activated by neurotransmitters, calcium ions, cellular swelling, and changes in transmembrane potential [89]. However, due to a lack of information, CLCN is well less understood than cation channels yet. The VGCLC family has nine different members which respond depolarization with an Cl^−^ inward flow at positive potentials, such as CIC-1 used in skeletal muscle, to maintain resting transmembrane potential. VGCLC plays a significant role in cellular survival, proliferation, colony formation, migration and malignancy [90, 91]. Within organelles, VGCLC also participates volume regulation, transport of anionic substrates, and electroneutrality remaining [92].

Although the ion channels-induced ion concentration is the dominant source of bioelectricity, there are additional factors that contribute to the generation of endogenous bioelectricity. For example, some biochemical substances, including amino acids, peptides, proteins, viruses, and polysaccharides also has piezoelectric property, which have been comprehensively reviewed by Wang et al. [46].

### The effects of bioelectric signals

#### Intercellular communication

Bioelectricity is considered a crucial signaling mediator which coordinates individual cell behaviors towards large-scale anatomical outcomes [93]. Previous part has demonstrated that cells regulate their own V_mem_ via ion channels, and this paragraph will illuminate how this V_mem_ propagate to and affect adjacent cells. The roles of gap junctions in V_mem_ propagation gradually emerged over the past decades [94]. They are widely expressed intercellular structures and the gap junctions in non-neural cells likely play a similar primitive function as synapses [95]. Chicken mesenchymal cells can propagate the bioelectricity to neighbors by gap junction and sonic hedgehog to coordinate their movement patterns [96]. Another interesting example is melanocytes. It was reported that calcium channel modulators can enhance or suppress pigmentation globally, but a gap junction inhibitor can change stripe patterning [97]. Meanwhile, it is well known that numerous carcinoma cells display diverse transmembrane potential from normal cell [98–100], and gap junction is as important for carcinogenesis [101]. Thus, some scholars proposed an interesting theory suggesting that malignant tumors use bioelectric signals to distinguish normal tissue from themselves and even affect the surrounding environment [95, 102]. Moreover, this propagation of bioelectricity is likely a non-neuron long-distance signaling mechanism, depolarization of instructor cells in the head is sufficient to influence melanocytes in the tail even though the mechanism remains unknown [103, 104].

On the other hand, bioelectric signaling coordinates multicellular behavior to guide organ-level geometry, regulating size and shape of organs [93]. More importantly, this signaling pattern is reprogrammable even in complex multicellular organisms. The wound in planaria exhibited significant depolarization 3 h after amputation. This depolarization triggered the downstream expression of head-specific genes, facilitating head regeneration. The bioelectric signaling pattern surrounding the wound can be disrupted by gap junction blockers or ion channel drugs. Depolarization applied to both ends resulted in the formation of mirrored two-headed worms, while the opposite change induced the development of no-headed worms [105, 106]. This suggests that bioelectric signals can electrically interconnect cells during repair, regeneration, and development, offering high-level instructions for patterning and morphogenesis.

#### Coupling of bioelectricity with mechanical force

Abundance studies demonstrated that mechanical stimulation plays an essential role in wide biological processes [107–111]. A variety of mechanical stimulation, fluid shear stress, tension, and (hydrostatic) compression, and matrix stiffness can modulate differentiation of stem cell [112], immune response [113], cellular apoptosis [114], tumor development [115] and bone remodeling [116]. An intimate connection between bioelectricity and mechanical forces has been acknowledged, wherein intracellular bioelectric signals and mechanical signals can reciprocally transfer. Cells sense mechanical stimulation through various mechanisms, with mechanosensitive ion channels (MSCs) serving as the primary cellular mechanical sensors expressed across all organisms and tissues, including the Piezo ion channel family [117]. This family includes two members (Piezo 1 and Piezo 2), which are nonselective cationic mechanosensitive channels. Piezo 1 was first identified in a neuronal cell line but subsequently confirmed to be presented in numerous mammalian tissues with particularly high expression in lung, bladder, and skin [118, 119] Under mechanical stimulation, Piezo 1 permits inward cationic ion influx, leading to depolarization, and becomes inactivated when the transmembrane potential reaches zero mV [117]. It has been demonstrated that Piezo1 not only converts mechanical signals into electrical signals but can also be directly influenced by changes in the transmembrane potential [120]. This suggests that Piezo1 acts as a pivotal node bridging mechanotransduction and electrical signaling. The two-pore domain potassium channels, specifically K2P (TREK-1), and the transient receptor potential vanilloid (TRPV) family represent two additional types of mechanosensitive ion channels that exhibit similar mechanosensitive mechanisms to Piezo1 [121–123]. In addition, calcium ions play a pivotal role as a common link between mechanotransduction and bioelectric signaling. Calcium ions participate mechanotransduction by altering their cytosolic concentration and subsequently activating downstream factors, including nuclear factor of activated T cells (NFAT), mitogen-activated protein kinase (MAPK), and calmodulin [124, 125]. Conversely, electric fields can directly regulate intracellular Ca^2+^ concentrations, thereby altering cellular mechanical properties [126]. Another possible coupling mechanism is that electric stimulation promotes filamentous actin polymerization and redistribution [127, 128], which is associated with cellular deformation and directed migration [129, 130].

#### Cellular migration

Since Emil Du Bois-Reymond first recorded the endogenous current in a wound [131], it has been known that the healing wound exhibits cathodic characteristics [132]. In wounded skin, a current from surrounded normal skin to wound center was generated, which offers directional cues for the cells [131]. This endogenous field emerges promptly upon the formation of a wound, operates prior to the establishment of chemical signals, and persists until the wound is completely covered by the epithelium [133, 134]. It suggests that the endogenous bioelectricity runs through the entire process of wound healing and compensates for the absence of chemical signals in the very early stage. Interestingly, this field can control the orientation of mitotic spindles in proliferating epithelial cells, causing them to divide parallel to the wound edge and perpendicular to the electric field vector [135]. Furthermore, the directional cellular migration induced by endogenous electric field is also observed in bone fracture [136], spinal cord injury [137] and early embryonic development [138]. However, cellular electrotaxis can be various based on different cell type. Specifically, neural crest cells, fibroblasts, keratinocytes, chondrocytes, rat prostate cancer cells, and many epithelial cell types migrate to cathode. However, corneal endothelial cells, bovine lens epithelium, human granulocytes, and human vascular endothelial cells trends to anode. In the electrical field generated by constant current, osteoblasts migrate toward the cathode and osteoclasts goes to opposite [139]. This diverse electrotaxis suggests the potential use of unidirectional current to steer directional bone remodeling in tissue engineering. There are three mechanisms of cellular electrotaxis: (1) the simplest mechanism is cell electrophoresis, charged cells migrated towards cathode or anode under the influence of electric field. Besides, cellular physicochemical component directly dictates electrophoretic mobility which is considered as an important marker indicates cellular biological state, such as drug resistance of cancer and function of red blood cell [140, 141]; (2) The endogenous electrical field induces cellular migration by modulate chemokines and chemokines receptors [142–144]. Luo et al. suggested a hypothesis: electric fields generate chemokine gradients, offering directional cues for cells [131]. This hypothesis is intriguing, particularly considering the charged nature of chemokines, even though no direct evidence has been reported; and (3) The endogenous electrical field results in redistribution of intracellular ions, proteins, and structures, which motivate cell to migrate along the direction of current. Electric field-induced depolarization occurs in the rear end of cell movement, then a Ca^2+^ influx and a Ca^2+^ wave to the front end [145]. In addition, Na^+^/H^+^ exchanger (NHE) isoforms located in the cell membrane and intracellular organelles are also involved. Under the influence of an electric field, phosphorylated NHE3 assembles at the leading edge of the cell, forming complexes with PKCη and γ-tubulin that are essential for directional cell migration [145]. As a result, H^+^ bubbles and β-actin are mustered at the leading edge, contributing to migration [146]. Collectively, the electric field can significantly facilitate cell migration even though the rate and direction of migration can be diverse.

#### Cellular proliferation

Bioelectric voltage is significantly associated to cellular proliferation. The actively proliferating cells usually has higher depolarization level, conversely, terminally differentiated somatic cells tend to display hyperpolarization. Bhavsar et al. inhibited depolarization of bone marrow-derived stem/stromal cells (BMSC) by pharmacologically blocking ion channels, leading to a successful reduction of proliferation [19]. In contrast, sustained depolarization was able to induce DNA synthesis and mitosis in mature neurons [147]. For cells with the strongest proliferative capacity, such as cancer cells, transmembrane potential is much more positive than in normal cells. This abnormality in transmembrane potential is associated with the aberrant expression of ion channels and transporters, contributing to various stages of the cancer process, including cell proliferation, apoptosis, migration, and invasiveness [148]. For an instance, the triple-negative breast cancer (TNBC) patients have overexpressed K^+^ channels (Kv1.5 or Kir2.1), causing more positive V_mem_ [22]. Thus, there have been ongoing efforts to utilize transmembrane potential as an early diagnostic marker or a target for drug development [23, 149, 150]. In addition, it has been reported that bioelectricity is employed in the electrostatic and magnetic capture of circulating tumor cells from whole blood [151]. Lastly, the detailed mechanisms of cellular proliferation induced by bioelectricity are still debated. Since cellular bioelectric state is a result of multiple factor (including various ion channels and extracellular environment), and many of these factors directly interact with cellular proliferation. Meanwhile, cellular proliferation is up to a bunch of biological elements, which make it more challenging to illuminate the mechanisms. Though the bioelectric regulation of cell cycle has been acknowledged [152], a comprehensive understanding of the intricate mechanisms necessitates further investigation.

#### Immune regulation

The immune regulation induced by bioelectricity is an excited topic. The positive impact of bioelectricity on wound healing has sparked scholars’ interest in its potential effects on preventing infections and inflammation. Exogenous direct-current stimulation applied to rabbit wounds exhibited a duration- and intensity-dependent antibacterial effect [131]. Nevertheless, intense direct-current stimulation can result in thermal injury to the host, which explains why certain studies have reported that intense electric stimulation yields poorer therapeutic outcomes than milder approaches [153]. In contrast, the immune regulation is a much more elaborate application of bioelectricity. Excited sympathicus can recruit immune cells to dental pulp, and the electric tooth stimulation can raise this effect [154]. Paré et al. used glycine receptor chloride (GlyCl) channels activator to reduce the transmembrane electric gradient (depolarization), enhancing *X. laevis* embryos’ resistance to infection [155]. Furthermore, potassium channel blocker, another typical intervention inducing depolarization, has a similar anti-infection effect. It suggests that this infection-resistance was not specific to a type of ion, but due to alteration of V_mem_. The exact mechanisms are still unclear, might involves serotonergic signaling and melanocyte-stimulating hormone (MSH) action. In conclusion, bioelectricity has an important impact on the regulation of the immune system, but the exact mechanisms are still ambiguous due to the limited number of studies.

## Exogenous electric stimulation

### The parameters

Electrical stimulation (EStim) therapy has a long history in treating diseases. Ancient Egyptians and Greeks were known to use electric eel to treat pain and various ailments by applying the shocks to the body. In 1831, the first electric generator in history was invented. Following that, from the mid-1800s to the early 1900s, exploiting people’s fear and curiosity about electricity, numerous charlatans claimed that their ‘electric therapy devices’ could treat almost all known diseases. Abundance invalid cases even fatality incidents impeded the development of electric stimulation therapy. Since the 1930s, with the advancement of modern medicine, EStim therapy has been substantiated as an effective approach for treating severe mood and psychotic disorders[156], bone fracture [157], and various neuromuscular pain [13, 14, 158]. However, some clinical trials and fundamental research have yielded diverse conclusions regarding EStim therapy [159–161]. This could be due to the high heterogeneity in the parameters of electrical stimulation used in these studies. The range of transmembrane potential changes is typically at the millivolt level [162], immortal breast cancer cells (− 30 mV) can exhibit extremely different genes transcription and cellular phenotype from normal breast epithelial cells (− 60 mV) [163]. In other words, even minor changes in EStim can lead to alterations in individual cell behavior, and achieving good repeatability requires extremely precise control of external EStim parameters. Moreover, even when exposed to the same EStim, different cell types may exhibit diverse responses. This variability can be attributed to the presence of distinct voltage-gated ion channels, charged components, electric resistance and initial transmembrane potentials within cells. As mentioned earlier, for example, osteoclasts and osteoblasts migrate in opposite directions when subjected to the same electric field [19]. Therefore, this paragraph aims to summarize and discuss the parameters of EStim employed by existing publications, offering the guide for determining optimal EStim protocol in the future research.

#### Type of electric stimulation

The clinically employed EStim technique comprises three categories: current stimulation, capacitive coupling (CC), and inductive coupling (IC).

**Fig. 3:**
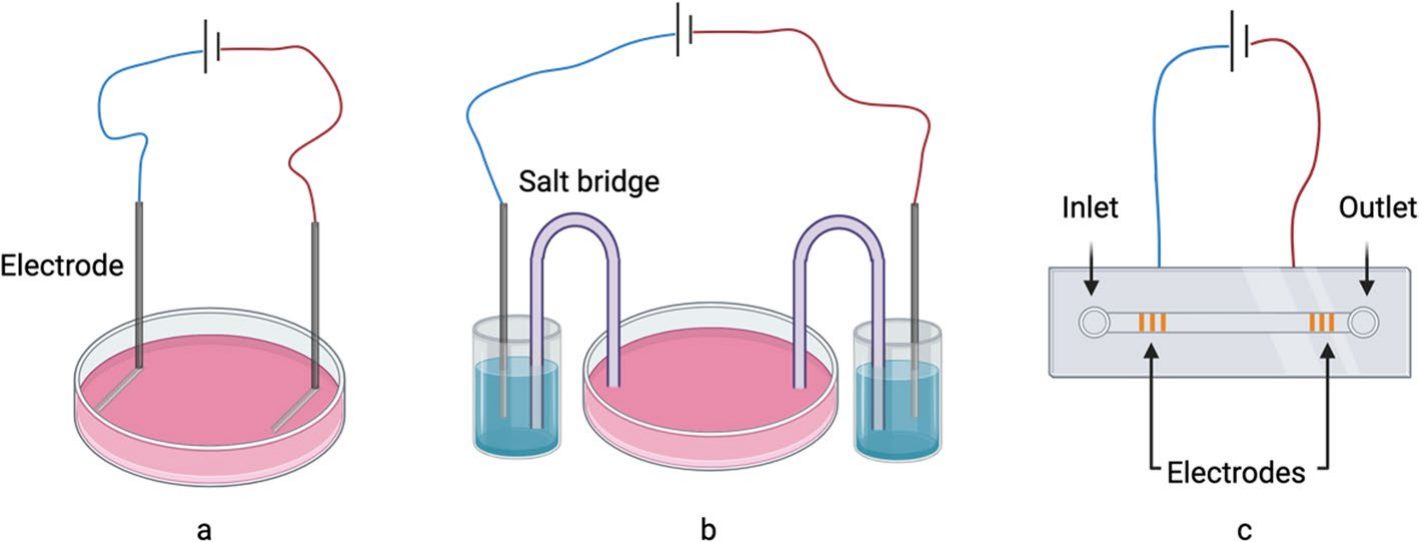
Three different direct-current EStim systems. **a** Direct current is applied between two metallic electrodes. **b** Two agar salt bridges connect culture medium to saturated solution of inert salt and delivery current. This system prevents cells from exposing directly to faradic products. **c** Microfluidic EStim chamber consist of a pair of electrodes, an inlet, an outlet and a fluidic channel. Cells are loaded through the inlet, driven to middle of the electrodes, exposed to EStim, and driven to outlet. The small cross-section of channel reduces the amount of toxic products

The current stimulation system is comprised of two electrodes, and it allows for the application of both direct current (DC) and alternating current (AC) between these electrodes. DC EStim system is the most commonly used model in vitro experiment due to its straightforward device and rapid accumulation of electric charge [164]. It is noteworthy that the electrochemical by-products (such as chlorine, hydrogen peroxide and reactive oxygen species) can inhibit the bacterial cells in vivo, but it interferes experimental result in vitro [165]. Therefore, salt bridge EStim chamber and microfluidic chip EStim chambers are two modified models which prevent cells from exposing directly to by-products (Fig. 3). But these models are all based on 2-dimensions cellular cultivation system, which cannot replicate completely the electric current and cellular behavior in 3-dimensions tissue. Related research based on 3-dimensional cellular cultivation is still necessary [165]. DC EStim has been applied in human and animals to regulate cortical excitability [166], promote osteogenesis [167], and guide cellular migration [168]. However, considering the potential damage from thermal accumulation and faradic product, the intensity and duration of DC applied on animal should be controlled strictly. AC EStim is a similar technique to DC but generates AC, and it is used frequently to modulate brain function [169, 170] and reduce pain [171]. In AC EStim system, the cathode and the anode periodically exchange with each other. This prevents the accumulation of oxidation or reduction products around a single electrode, which is its advantage compared to DC. Besides, AC was believed to mimic endogenous signal than DC, but there is no evidence to support that AC performs better in medical therapy [83]. Clinically, though both DC and AC can be administered by non-invasive electrodes, they are only performed in the electricity sensitive neuromuscular tissue, such as transcranial current stimulation and transcutaneous spinal direct-current stimulation [172, 173]. Since current generated by non-invasive electrode can hardly reach the deep target [174], the invasive electrodes are recommended for the tissue with higher electric resistance, such as bone, whereas patients may experience additional surgeries and an increased potential for infection due to the involvement of implantable electrodes. Thus, invasive DC or AC EStim are primarily utilized for those patients who require metal implants such as dental implants or fracture fixation nails.

The continuous current stimulation may lead to some side effects, such as thermal damage or chemical burning [175]. Implanted electrode can also provoke immune response or infection. Consequently, some scholars have suggested non-invasive and noncontact electric field stimulation techniques, including capacitive and inductive coupling electric field stimulation. Capacitive coupling entails the non-invasive placement of two cutaneous electrodes on opposite sides of the wound to be stimulated but affects deeper tissues compared to invasive DC. Capacitive coupling has been validated as an effective method of physical stimulation for enhancing spinal fusion [176], fracture union [177], and wound healing [175]. It is noticeable CC also generate heats in the deep tissues. It has been utilized to heat the deeper layers of the skin to promote collagen production for cosmetic purposes [178]. Next, pulsed electromagnetic field (PEMF) is the most widely employed inductive coupling technique due to its non-invasive and safe characteristics. This technique utilizes external current-carrying coils, driven by a signal generator, to generate a magnetic field within deep tissue, which has been approved for fracture healing and spinal fusion [176].

Since investigations into the three EStim types involve various variable values, such as targeted tissues, frequency, intensity, and durations, it is challenging to determine the most effective one through direct comparisons of these studies. Only a meta-analysis compared them and demonstrated that there is no significant difference among effects of three techniques on spinal fusion [179]. Therefore, when choosing among the three electrical stimulation methods, the primary consideration should be whether the chosen stimulation mode is suitable for the target site and acceptable to the patients, rather than solely focusing on their effectiveness.

#### Electrode polarity

Numerous investigations have reported that cathode and anode can elicit completely different tissue responses. For an instance, the cathode stimulation can favor the fracture healing, but the anode has adverse impact on healing [180]. The first reason is that cellular galvanotaxis is diverse, dependent on species and/or cell subtype. Most cells migrate towards cathode; this phenomenon may be attributed to the negative transmembrane potential of these cells. The side of the membrane facing the anode hyperpolarizes and attracts free calcium ions, leading to membrane contraction and propelling the cell towards the cathode [181]. However, some cells migrates to anode, such as osteoclast [139] and adipose-derived stem cells [28] and human vascular endothelial cells [182]. Mycielska et al. believed that this abnormal galvanotaxis was associated with activation of voltage-gated cathode facing cathode [181]. Second, cathode stimulation more easily induce cellular depolarization than anode, initiating different signaling pathways [183]. Lastly, the faradic by-products at cathodes are different from those at anodes. The cathode can produce reactive oxygen species (ROS) and raise pH value, both of which increase osteoblast activity [176, 184]. In contrast, the anodic reaction makes the metal electrode dissolve and releases toxic ions [185]. Thus, it is crucial to decide the site of electrodes and current direction according to the target cell/tissue type and purpose. Considering injured skin as an example, a short-circuiting of the transepithelial potential results in a more negatively charged wound compared to the surrounding tissue [91]. When a fracture occurs, fracture site also turns negatively charged [47, 186]. The current from intact tissue to wound accelerates recruitment of immunocytes and cytokines, contributing to inflammation and tissue repairment [91]. Therefore, this negative potential presented in the wound is considered a signal for ‘healing’. It explained why the cathode should be placed in the wound center and anode should be in the intact tissue.

#### Intensity of stimulation

All methods of EStim appear to raise the intracellular calcium level to stimulate cellular proliferation [187, 188]. However, excessive calcium can lead to cellular apoptosis [22]. Therefore, it is essential to investigate the appropriate intensity of electricity stimulation for EStim application. Yet, there is no clear consensus on the optimal intensity because associated literatures are highly heterogeneous (Table 2). Even the units used for evaluating the magnitude are debated. For current stimulation, volt (V) and ampere (A) are two common units, representing constant voltage stimulation and constant current stimulation, respectively. Christian et al. believed constant voltage stimulation can reduce the potential tissue damage compared to constant current, but another study found the difference to be minimal [189, 190]. Furthermore, volt per meter (V/m) and ampere per square meter (A/m^2^) were considered as better units to evaluate intensity because they considering the volume and cross-section area of tissue [191]. In the context of PEMF, Gauss is the most frequently used unit to describe the magnitude of EStim [192, 193]. Hence, in future research, it is imperative to establish a universally recognized unit for describing the magnitude of electric fields within tissues.

**Table 2:** A decade of literature on the utilization of the EStim system and their comprehensive parameters

Tissue or cell	Electrical Type	Intensity	Pulse	Frequency	Duration	Pulse width	Wave form	Outcomes	Refs.
Rat (L3 and L4 dorsal root ganglions)	Current stimulation (DC)	4 V/cm	Yes	Unmentioned	20 min/day; last for 56 days	500 μs	Square	Accelerated femoral osteoporotic fracture healing	[157]
Sheep (pelvic limb)	Current stimulation (AC)	1500 μA	yes	60 kHz	12 h/day, last for 30 days	40 ms	Unmentioned	Accelerated fracture healing	[199]
Sheep (dental implant–bone interface)	Current stimulation (DC)	7.5 μA	No	–	6 h/day, last for 84 days	–	–	No detectable improvement	[200]
Human (mesenchymal stem cells)	Capacitive coupling	1–2 V/cm	No	–	Continuous stimulation, last for 14 days	–	–	Osteogenic differentiation and increased calcium deposition	[194]
Rat (hindlimb skeletal muscle)	Current stimulation	1.5 V	Yes	40 Hz	60 min/day, 5 days/week, last for 28 days	Unmentioned	Unmentioned	Reduced muscle atrophy	[201]
Human (chondrocytes)	Capacitive coupling	2–3.5 V/cm	Yes	1 kHz	45 min × 3 times /day, last for 7 days	Unmentioned	Unmentioned	Increased genes expressions of collagen type II and aggrecan	[202]
Human (calvarial osteoblasts)	Capacitive coupling	20 mV/cm	Yes	60 kHz	1 h	Unmentioned	Unmentioned	Increased genes expressions of bone morphogenetic proteins (BMP)-2 and -4, TGF-β1,—β2 and -β3, fibroblast growth factor (FGF)-2, osteocalcin (BGP) and alkaline phosphatase (ALP)	[203]
Human (mesenchymal stem cells)	Current stimulation (DC)	1 V/cm	No	–	10 min/day, last for 14 days	–	–	Early osteogenesis in hMSCs; increased reactive oxygen species	[198]
	Current stimulation (AC)	1 V/cm	Yes	10 Hz	10 min/day, last for 14 days	100 ms	Square	Late osteogenesis	
Mouse (bone marrow-derived macrophages)	Current stimulation (AC)	0.5 V	Yes	500 Hz	6 h/day, last for 3 days	Unmentioned	Square	M1 polarization	[197]
	Current stimulation (AC)	0.5 V	Yes	500 Hz	6 h/day, last for 3 days	Unmentioned	Sinusoidal	M2 polarization	
Human (chondrocytes)	Current stimulation (DC)	1.5 V	No	–	1.5 h/day	–	–	Early late osteogenesis	[167]
Human (dermal fibroblasts)	Capacitive coupling	54 V/cm	Yes	60 Hz	2 h/day	100 μs	Unmentioned	Increased proliferation and migration	[175]
Human (keratinocytes)	Capacitive coupling	54 V/cm	Yes	60 Hz	2 h/day	100 μs	Unmentioned	Increased proliferation and migration	
Mouse (skin wound)	Capacitive coupling	54 V/cm	Yes	60 Hz	2 h/day	100 μs	Unmentioned	Accelerated wound healing	
Mouse	Inductive coupling (PEMF)	146.7 Gauss	Yes	75 Hz	Continuous stimulation, last for 5 days	–	Unmentioned	inhibition of inflammatory cytokines	[192]
Human (teared supraspinatus tendon)	Inductive coupling (PEMF)	25 Gauss	Yes	50 Hz	25 min/day, 5 days/week, last for 14 days	Unmentioned	Unmentioned	No detectable improvement	[204]
Mouse (swelled paws)	Inductive coupling (PEMF)	145 Gauss	Yes	10 Hz	Continuous stimulation, last for 32 days	Unmentioned	Unmentioned	Reduced local inflammatory cytokines and bone destruction	[193]
Rat (osteochondral defect)	Inductive coupling (PEMF)	Unmentioned	Yes	Unmentioned	3 h/day, last for 14 days	Unmentioned	Unmentioned	Chondrogenesis and cell hypertrophy via ERK and p38 MAPK pathways	[205]
Mouse (melanoma B16F10)	Inductive coupling (PEMF)	400 A	Yes	80 Hz	Unmentioned	Unmentioned	Unmentioned	Electroporation; Enhanced uptake of platinum	[206]
Rat (cardiac tissues)	Current stimulation (AC)	5 V	Yes	1 Hz	1 h/day, last for 3 days	10 μs	Square	Increased oxygen consumption and membrane permeability; Decreased contraction frequencies	[196]
Human (mesenchymal stem cells)	Capacitive coupling	1 V/cm	Unmentioned	Unmentioned	2 h/day, last for 21 days	Unmentioned	Unmentioned	Osteogenesis	[207]
Human (palatal wound)	Current stimulation (AC)	100 μA	Yes	9 kHz	30 s/day, last for 3 days	Unmentioned	Unmentioned	Positive effect on early wound closure; reduced inflammatory cytokines	[208]
Mouse (skin wound)	Capacitive coupling	53 V/m	Yes	Unmentioned	Continuous stimulation, last for 12 days	Unmentioned	Unmentioned	Reduced scarring, enhanced collagen synthesis; increased angiogenesis	[195]

The duration of exposure to EStim also dictates the intensity of stimuli. Many studies have employed intermittent stimulation once a day, primarily due to concerns regarding potential damage from EStim, particularly with current stimulation. Only a few studies have explored continuous stimulation using low-magnitude capacitive or inductive electrical fields [194, 195]. Functional and molecular alterations can typically be observed as early as 3 days after EStim initiation [196, 197], while the histological changes often require a more extended timeframe [195, 198]. In conclusion, the intensity of stimulation is depended to EStim types, magnitudes and durations, and the optical intensity may largely vary depending on the target tissue. More details can be found in Table 2.

#### Pulsed electric stimulation

In contrast to static electric stimulation, pulsed electric stimulation leads to different cellular reaction [209]. For pulsed EStim, three parameters should be considered: frequency, waveforms and pulse width. First, low-frequency pulsed current (5 Hz) elicits the more severe muscular fatigue than high-frequency (75 Hz), but it is easier for muscle to recover from the fatigue induced by low-frequency stimulation [210]. It was also reported that high-frequency stimulation can stimulate cell to release more anabolic signal and cytokines [211, 212]. Second, rectangular and sinusoidal pulse shapes are two fundamental waveforms most frequently used in pulsed EStim [213]. Transcranial magnetic stimulations with rectangular wave cause greater cortical inhibition than those with sinusoidal waves [214], but there is no difference between effects of two waveforms on facial muscles [215]. In addition, square waveform promoted M1 polarization but the sinusoidal waveform promoted both M1 and M2 polarization [197]. Wave width stands out as a critical parameter in electroporation. A pulsed electric field with nanosecond-level pulse width can create significantly smaller pores on the cytomembrane compared to those observed with microsecond-range electroporation, which only permits the passage of small molecules like ions [216]. Hence, some biological effects, such as cell apoptosis induced by electroporation, can only be triggered by EStim with definite wave width [217]. In summary, despite a number of clinical research revealed the significance of parameters of pulsed EStim, the underline mechanisms still require more investigations before complete emerging.

### Applications in dental medicine

#### Diagnosis and therapy of endodontic disease

Electric stimulation has been used to assess the condition of the nerves within the dental pulp [218]. However, this assay method may not always accurately reflect the actual state of dental pulp, as immature teeth or teeth with temporarily disable the sensory nerves can exhibit false-positive or false-negative responses [219, 220]. The site where the probe is placed and adjacent restorations also can interfere the assay results [221]. At present, some scholars have explored the laser Doppler flowmetry as the next-generation method for determining dental vitality. This approach assesses pulpal blood flow rather than nerve fibers, offering a promising alternative [210].

Another example of application is electric apex locator, which utilizes high-frequency microcurrent and records electric impedance between canal and periodontium to measure the canal’s working length and locate the apex constriction. The accuracy of measurement is essential for the success of endodontic procedures [222].

Besides, some studies tried to conduct bone diagnosis monitor using electromechanical impedance technique [223]. An external piezoelectric transducer was used to apply the high-frequency vibration on tooth, effectively transmitting these vibrations into the deeper bone tissue. The vibration of bone, which are influenced by its inherent, were subsequently recorded using a piezoelectric patch. This non-invasive method serves as a means to detect bone density and can also be utilized to assess the osteointegration of dental implants [224]. However, it is still uncertain whether these vibrations may adversely affect the osteointegration of implants, especially those with poor stability. Thus, more clinical trials are still necessary.

#### EStim coupled with dental biomaterials

Considering that electric stimulation has demonstrated its potential to enhance bone fracture healing in animal and clinical experiment, its application in maxillofacial surgery has gradually emerged over the past decade. While exogenous electric stimulation usually requires an external electric source and wires, these devices connected to the maxillofacial surgical area can interfere with patients’ daily activities and reduce compliance [159]. Even though those devices can be intraorally placed, the oral environment may cause the electric source corruption and the release of toxic chemical substances. A promising solution is coupling the EStim with tissue engineering [225]. A predictable approach involves applying the EStim to treat cell-scaffold constructs before implantation, which can greatly improve outcome in tissue engineering treatments. Bueno found that xanthan/polypyrrole scaffolds treated with EStim favored cell adhesion [226]. Similarly, Cheng et al. successfully employed 0.33 V/cm electric field to enhance osteo-differentiation of human dental pulp-derived stem cells on the polypyrrole (PPy) films [227]. These modified biomaterials hold potential applications in the maxillofacial surgery and dental bone augmentation.

On the other hand, a wealth of piezoelectric biomaterials has been reported [228–232], capable of self-generating long-term electric stimulation from within tissues. These piezoelectric biomaterials usually have highly ordered crystal lattices. When subjected to mechanical-stress-induced deformation, a relative shift of the positive and negative charge center within the material crystal structure occurs, resulting in motion of an electric dipole or polarization [233]. In simpler term, these materials can convert the mechanical stress from physiological activities into the electric current. These materials, including barium titanate (BT), boron nitride (BN), zinc oxide (ZnO), hydroxyapatite (HA), poly(vinylidene fluoride) (PVDF), poly(vinylidene fluoride-trifluoro ethylene) (P(VDF-TrFE), gallium nitride (GaN), lithium niobate (LN), lithium sodium potassium niobate (LNKN), potassium sodium niobate (KNN), have been widely investigated for various biomedical applications [234]. Besides utilized in bone tissue engineering [235], the piezoelectric material can be incorporated to dental composite as nanofillers. Montoya et al. developed a novel multifunctional dental composite with barium titanate (BaTiO3) nanoparticles which serves antibacterial and mineralization roles [236]. Furthermore, piezoelectric biomaterials can be harnessed to offer energy for photo-biomodulation therapy. Park et al. reported on an implant which uses barium titanate piezoelectric material to harvest energy from human chewing and brushing, supplying energy to a red LED [237]. At last, Carter et al. proposed the use of converse piezoelectric materials, which deforms under electric stimulation, suggesting these materials can provide beneficial mechanical stimulations in areas where mechanical loading has decreased or stress shielding has occurred [238]. Nonetheless, the potential for this micro-motion of converse piezoelectric materials to cause mechanical damage to the surrounding tissue raises uncertainties, which could hinder its broader application.

#### Orthodontic tooth movement

Orthodontic tooth movement (OTM) is a process of alveolar bone remodeling inspired by compressive force. Due to the piezoelectric properties of bone, electric signal is considered as one of initiating factors of OTM [239]. Consequently, some scholars have explored the potential of EStim as an adjunctive intervention to accelerate OTM. Spadari et al. reported that a 10 μA current increased the number of osteoclasts and enhanced vascularization during OTM in rats [240]. In addition, a 15 μA current was verified to up-regulate cAMP and cGMP in cats’ periodontal ligament [241], which are second messengers starting bone turnover [242]. However, these reports did not specify the tooth movement rate or other current parameters (direct/alternating, direction, frequency, waveforms). Besides, PEMF was reported to increase of 31% in the rate of canine retraction by a recent clinical trial including 19 patients [243]. A similar conclusion was reached by another trial conducted by Showkatbakhsh et al. [244], whereas the quality of these studies may not be sufficient to definitively support microcurrent or PEMF as effective adjunctive interventions. Further randomized control trials (RCTs) with larger sample sizes are still necessary before considering the clinical application of EStim.

Orthodontic pain management is another crucial aspect associated with patients’ compliance during orthodontic therapy. Orthodontic stress can excite primary somatosensory cortex (S1), the ventrocaudal part of the secondary somatosensory cortex (S2), and the insular oral region (IOR) in the brain through neural electric signal, leading to radiating pain [245]. A study based on rabbits revealed that electric acupoint stimulation can reduce algogenic substance PGE2 in peripheral nervous system and increase analgesic substance (endorphin) in central nervous system [246]. Moreover, two clinical trials also demonstrated that transcutaneous electric nerve stimulation can decrease orthodontic pain, even its effective seems to be lower than laser therapy [247, 248]. In a RCT involving on 32 female patients, it was shown that PEMF reduced orthodontic pain after 24 h [249]. While these pieces of evidence are more convincing than those suggesting EStim accelerates OTM, they still exhibit high heterogeneity in parameters and a high risk of bias. Further research investigating the mechanistic aspects of EStim as an analgesic therapy is still needed.

#### Osteointegration of dental implant

Pure titanium and titanium alloys are the most commonly used materials for orthopedic/dental implants [250]. They are ideal for implantable electrodes due to their excellent electrical conductivity. It is noticeable only currents exceeding 10 μA promote the osteointegration [251]. This might explain why some research yielded negative results when using insufficient microcurrent (7.5 μA) [200]. Pettersen et al. suggested pulsed current perform better than continuous current, as it mimics peripheral nerve stimulation. As a result, he recommended the 20 μA and 50 Hz as the optimal parameter to induce cell to adhesion to titanium plate and simulate collagen production [250]. The capacitive electric field of 3 V can increase bone formation and bone contact around implant in beagles [252]. Interestingly, some weaker pulsed currents (115 Hz, 1.68 μA/cm^2^) were found to inhibit the rate of osteointegration, although exact reason is unknown [253]. Another concern with EStim is its potential to cause corrosion of metal implants, with the released metal ions possibly affecting osteointegration, reducing implant mechanical strength, and impairing local tissue [254]. Future clinical trials should focus on long-term effect of EStim on osteointegration of implants.

#### Maxillofacial malignant disease

Previous research has shown that malignant cells exhibited different cell transmembrane potential compared to non-malignant cells [255]. This suggests that transmembrane potential could serve as a potential diagnostic or therapeutic target for malignant diseases. For instance, Yu et al. employed an engineered voltage-gated calcium channel that can be selectively activated in breast tumor to selectively kill breast tumor cells [23]. In the field of dental medicine, early studies attempted to treat maxillofacial carcinoma using high-voltage electrical impulses [256]. However, a significant drawback of this method was that electric impulses also cause severe damage to normal tissue. Later, nanosecond pulsed electric fields were reported to sensitize oral tongue squamous cell carcinoma to conservational radiation and chemical therapy [257, 258]. To achieve more selective and effective therapies, further research into the mechanisms and clinical trials examining the effects of EStim on maxillofacial malignant diseases are necessary.

#### Maxillofacial nerves

Previous studies have established the sensitivity of the neuron system to electrical stimulation (EStim). In the field of dental medicine, EStim has found application in various contexts, including the enhancement of facial nerve repairment [259], the stimulation of the hypoglossal nerve to address obstructive sleep apnea (OSA) [260], and the management of orofacial neurogenic pain [261–264].

Following an extensive literature review, only one animal study has been identified that reported the potential of electric stimulation in facilitating the repairment of the maxillofacial nerve. Mendez et al. [259] applied brief electrical stimulation (BES) and suggested its capacity to expedite preferential motor reinnervation. However, robust evidence in this context remains limited.

In contrast, numerous studies have validated hypoglossal nerve stimulation as a novel therapeutic approach for moderate and severe OSA [265–267]. Hypoglossal nerve stimulation involves the generation of electric impulses by an implanted chest skin-based generator. This electric stimulation of the hypoglossal nerve enhances the function of tongue protrudors and retractors, resulting in pharyngeal dilatation during expiration and greater airway stability [260].

The mechanisms behind the analgesic effects of electrical stimulation remain somewhat enigmatic. One theory postulates that EStim inhibits the transmission of pain signals through C fibers while exciting A fibers, thereby exerting presynaptic inhibition [268]. However, Nathan and Wall reported that EStim appears ineffective in treating severe post-herpetic neuralgia, attributing this ineffectiveness to damage to A fibers [269]. Unfortunately, due to the unclear underlying mechanisms of diseases like trigeminal neuralgia and orofacial pain, our understanding of electrical stimulation therapy primarily remains at the level of symptomatic treatment.

#### Maxillofacial muscles

Various forms of electric stimulation have been employed to manage maxillofacial muscular disorders and pain. First, two clinical trials conducted by Fagade et al. have demonstrated that 30-min sessions of transcutaneous electrical nerve stimulation (TENS) improved forced mouth-opening exercises in patients who had undergone trismus [270, 271]. Moreover, TENS has proven effective in reducing pain associated with temporomandibular joint disorders (TMD) [272].

Second, microcurrent nerve stimulation (MENS) is another frequently utilized electrical stimulation technique known for its efficacy in alleviating muscle myofascial pain induced by bruxism or TMD [273–275]. A randomized controlled trial by Saranya et al. revealed its superior effectiveness in pain relief when compared to TENS [273].

Lastly, percutaneous needle electrolysis (PNE) has been reported to rapidly reduce temporomandibular myofascial pain, as evidenced by a randomized controlled clinical study [276]. Despite the long-standing clinical use of electrical stimulation as a therapeutic modality, our understanding of its mechanisms for muscle analgesia remains limited. Therefore, further investigation into the cellular mechanisms involved is imperative.

## Future direction

In the early stages of bioelectricity discovery, it was primarily recognized as a physiological phenomenon influencing various life activities. During that time, electricity was a relatively novel concept, leading to public curiosity, fear, and even religious reverence towards it. Consequently, a multitude of electrotherapy devices entered the market without undergoing rigorous clinical trials, often with grossly exaggerated claims, resulting in severe physiological harm to patients in some instances. As our understanding deepened, we came to realize that bioelectricity is a result of differences in intra- and extracellular ion concentrations, dispelling notions of it being an occult phenomenon [277]. Advancements in molecular biology in the modern era allowed us to gain profound insights into bioelectricity. Figures such as Roderick Mackinnon, William Catterall, and Ardem Patapoutian made significant contributions in this area, unveiling the molecular-level processes through which charged ions traverse cell membranes and their impacts on physiological process [278–283]. Up to now, we have recognized that the bioelectricity is one of signal mediators, an important chain of biophysiological cascade. Bioelectrical signals not only affect individual cells but are also transmitted between cells following specific patterns. Michael Levin et al. proposed a groundbreaking perspective in which non-excitable tissues can harness bioelectric encoding of distributed goal states, akin to how the brain functions [93, 105]. This bioelectric pattern plays a fundamental role in the normal morphogenesis of multiple organs [284–287]. The future focus of research should center on decoding and reprogramming this bioelectric pattern, offering possibilities for intervening in physiological development and disease processes. These findings also lay the theoretical fundament for the development of Xenobot with complicated functions [288].

All types of electric stimulations, ion channel blockers, and even mechanical stimulations are all considered as interventions targeting this bioelectric pattern in some ways. In the field of dental medicine, these interventions have achieved some clinical success, as mentioned in the previous chapter. However, the primary limitation lies in the lack of consensus regarding the application methods and parameters of electrical stimulation, which may contribute to conflicting research findings. It is difficult to determine optimal methods and parameters because stimulation parameters used by existing publication is too heterogeneous to be referred by clinician. Meanwhile, resistance and endogenous bioelectricity can vary significantly in various tissue with different states. This leads to inconsistent alterations in the bioelectrical state, even when the same parameters of electrical sources are employed within various tissues of different individuals. Therefore, a non-invasive visualized tools for dynamically monitoring bioelectricity state is required to determine the optimal stimulation on the dental clinical practice. Another challenge is that most mechanism studies focus on single ion or single channel. But even at the level of individual cells, their bioelectric status results from the interaction of hundreds of ion channels. Similarly, the bioelectric state within tissues is an outcome of the interactions among various cell types. Thus, high-throughput electrophysiology techniques aid in enhancing our understanding of cellular electrophysiological regulatory mechanisms. On the other hand, currently, prevailing approach for modulating cellular bioelectricity relies on ion channel activators or inhibitors. Pharmacological modulation is subject to numerous influencing factors, leading to a lack of precision and reproducibility. It is necessary to generate more predictable bioelectric interventions. The ideal bioelectric modulation should be capable of modulating cell membrane potential at mV or even μV levels, thereby purposefully influencing tissue development, repair, and even regeneration. Lastly, the development of biocompatible and wearable nanomaterials is a crucial area for the future. Induced bioelectric changes represent an important category of effects to consider for bioengineers developing dental biomaterials that guide stem cell differentiation and promote regenerative tissue growth.

## Conclusions

A comprehensive understanding of endogenous bioelectricity has been gained. However, the therapeutic efficacy of exogenous electrical stimulation in organisms remains controversial, primarily due to the heterogeneity of existing research methods. Over the past decade, bioelectricity has begun to find applications in various domains of dental medicine. However, the existing literature primarily consists of case reports and small-scale trials. Heterogeneity in both EStim system and clinical outcomes is also a concern. Therefore, the imperative for large-scale, standardized, randomized controlled clinical studies persists. Furthermore, in the future, precise measurement and modulation methods of bioelectric signal patterns represent a critical research direction in this field.

## Data Availability

All data generated in study appear in the submitted article.
